# Supplementary material for: WGA-M001, a Mixture of Total Extracts of Tagetes erecta and Ocimum basilicum, Synergistically Alleviates Cartilage Destruction by Inhibiting ERK and NF-κB Signaling
Source: Int J Mol Sci. 2023 Dec 14;24(24):17459. doi: 10.3390/ijms242417459 (PMC10743532; doi:10.3390/ijms242417459)
Supplement: Supplementary file 1 [file ijms-24-17459-s001.zip › ijms-2713314-supplementary.pdf]

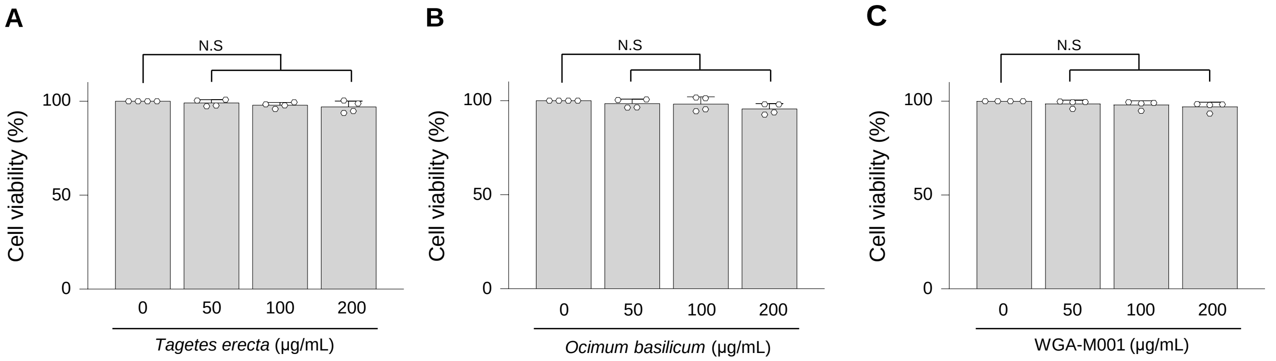

Figure S1. Toxicity of *Tagetes erecta* (A), *Ocimum basilicum* (B), and WGA-M001 (C) to chondrocytes. Cell viability was measured at various concentrations for 24 h and analyzed using a lactate dehydrogenase (LDH) assay. Data were analyzed using one-way analysis of variance with Bonferroni's test, and the plotted values are indicated as means ± standard error of the mean.

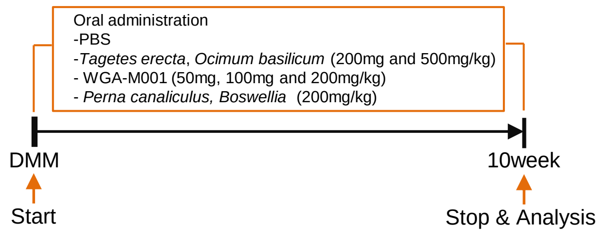

Figure S2. Oral administration plan for analysis of DMM-induced arthritis. Mice were administered phosphate-buffered saline (PBS) or indicated substances every day after DMM surgery until analysis.
